# Toxicity Assessment of Silica Coated Iron Oxide Nanoparticles and Biocompatibility Improvement by Surface Engineering

**DOI:** 10.1371/journal.pone.0085835

**Published:** 2014-01-21

**Authors:** Maria Ada Malvindi, Valeria De Matteis, Antonio Galeone, Virgilio Brunetti, George C. Anyfantis, Athanassia Athanassiou, Roberto Cingolani, Pier Paolo Pompa

**Affiliations:** 1 Center for Biomolecular Nanotechnologies@UNILE, Istituto Italiano di Tecnologia, Arnesano, Italy; 2 Nanophysics, Istituto Italiano di Tecnologia, Genova, Italy; 3 Istituto Italiano di Tecnologia, Genova, Italy; Brandeis University, United States of America

## Abstract

We have studied *in vitro* toxicity of iron oxide nanoparticles (NPs) coated with a thin silica shell (Fe_3_O_4_/SiO_2_ NPs) on A549 and HeLa cells. We compared bare and surface passivated Fe_3_O_4_/SiO_2_ NPs to evaluate the effects of the coating on the particle stability and toxicity. NPs cytotoxicity was investigated by cell viability, membrane integrity, mitochondrial membrane potential (MMP), reactive oxygen species (ROS) assays, and their genotoxicity by comet assay. Our results show that NPs surface passivation reduces the oxidative stress and alteration of iron homeostasis and, consequently, the overall toxicity, despite bare and passivated NPs show similar cell internalization efficiency. We found that the higher toxicity of bare NPs is due to their stronger *in-situ* degradation, with larger intracellular release of iron ions, as compared to surface passivated NPs. Our results indicate that surface engineering of Fe_3_O_4_/SiO_2_ NPs plays a key role in improving particles stability in biological environments reducing both cytotoxic and genotoxic effects.

## Introduction

Iron oxide nanoparticles (IONPs) naturally form as nano-sized crystals in the earth's crust. They are also abundant in the urban environment, especially in underground stations [1]_ENREF_1, railway lines [2], or at welding workplaces [3]. Furthermore, in recent years, their unique magnetic properties have shown great potential in various biomedical applications for both diagnosis and therapy, such as contrast agents in magnetic resonance imaging (MRI) [4]–[6], drug [7] and gene delivery carriers [8] and cancer hyperthermia [9]. The widespread presence and the therapeutic benefits of IONPs, however, raise concerns about their toxicity. Therefore, understanding the potential hazard and the physico-chemical parameters underlying toxicity of IONPs is crucial. Even though IONPs have already been used in clinical applications [10], [11], the literature shows conflicting results about their toxicity [12], [13]. Systematic studies on their cytotoxic effects are rare, and often affected by insufficient characterization and short-term evaluation of their cellular impact. Several approaches focused on the encapsulation of magnetic nanoparticles with different materials to improve their biocompatibility, namely: dextran [14], [15], silica [16], [17]_ENREF_14, chitosan [18], and polyethylene glycol [19]. However to date the role of surface coating is not yet clear. Some studies speculated that iron oxide nanoparticles could be degraded into iron ions within the lysosomes after cell internalization [20], [21]. The chemical synthesis, as well as the presence and the physico-chemical properties of the coating, which surrounds and isolates the magnetic material from the environment, may influence the degradation rate of the particles and so the release of iron ions [21], [22]. The nanoparticles degradation process in lysosomes begins with the degradation of the corona that adsorbs on the nanoparticles and continues slowly with the particles core [23]. Hence, understanding the relationship between iron ions release from the nanoparticles and cell toxicity is important to better understand IONPs toxicity and their long term effects, as well as to design safer nanosystems exploitable for biomedical applications *in vivo*. In this study we evaluate the effect of NPs surface passivation on toxicity mechanisms. Monodispersed and stable iron oxide nanoparticles covered with a thin silica shell (Fe_3_O_4_/SiO_2_ NPs) were tested. Silica is known to be one of the most suitable coating layer for superparamagnetic NPs due to its chemical stability, biocompatibility [24], [25], versatility for surface modification. Moreover, it helps to convert hydrophobic NPs into hydrophilic water-soluble particles [26]. We evaluated both bare and surface passivated Fe_3_O_4_/SiO_2_ NPs with different chemical functionalizations. The passivation was done with amino- or sulfonate- silanes, respectively producing NPs with positive and negative surface charges. The toxicity assessment was performed in two cell lines (A549 and HeLa), using five different toxicity tests: WST-8 assay (cell viability), LDH assay (cell membrane integrity), DCF assay (ROS levels), mitochondrial membrane potential (MMP) assay, and comet assay to evaluate DNA damage. We then focused on their toxicity mechanism, evaluating the influence of surface engineering on the release of iron ions, and their effects on the cell toxicity.

## Results and Discussion

**Figure 1:**
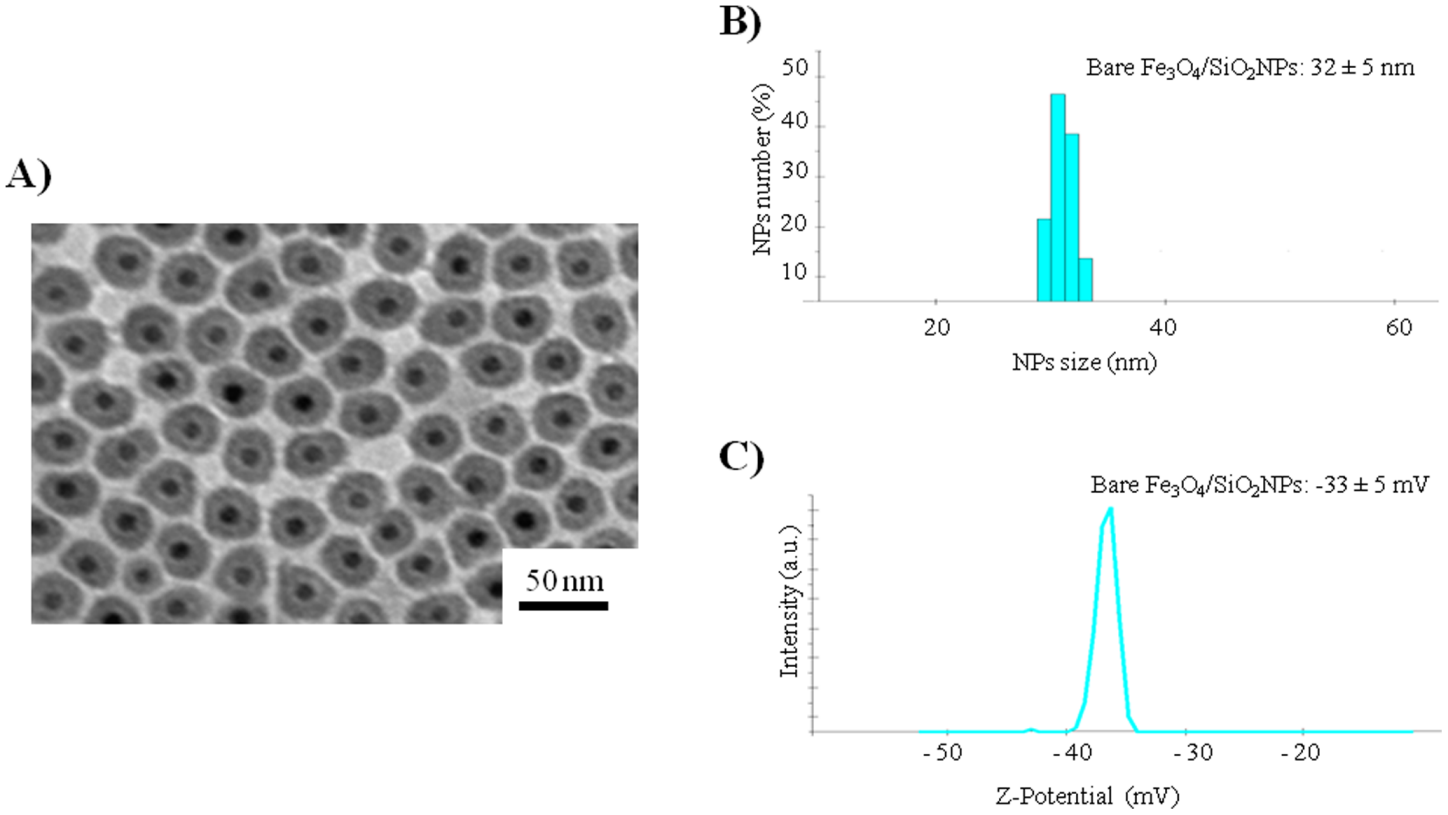
Characterization of bare Fe_3_O_4_/SiO_2_ NPs in water. (A) TEM image, (B) Dynamic light scattering, (C) ζ-Potential measurements.

**Figure 2:**
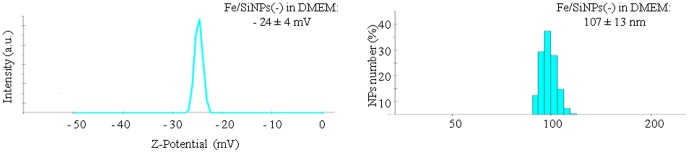
Characterization of bare Fe_3_O_4_/SiO_2_ NPs in cell culture medium. (A) ζ-potential and (B) Dynamic light scattering after 96 h incubation in DMEM culture medium.

Three types of monodispersed Fe_3_O_4_/SiO_2_ NPs were evaluated: bare, amine- , and sulfonate- silane surface modified NPs. The aim was to determine whether the NPs surface coating provides increased stability, reducing adverse cellular effects, regardless of the NPs surface charge. Fe_3_O_4_ NPs were obtained through a colloidal synthesis (see Experimental). Then, Fe_3_O_4_ NPs were coated with a thin silica shell by the microemulsion method [27]. Bare NPs, exposing OH groups, were obtained directly from the microemulsion synthesis, while surface passivated NPs were functionalized with two different groups on their surface. Our goal was to create passivated NPs with identical size and surface chemistry, but different surface charge. We thus chose two molecules: aminopropyltriethoxysilane (APTES) and (trihydroxysilyl)-1-propanesulfonic acid (SIT) with similar chemistry structure, but different functional groups. APTES was used to produce the aminosilane coating (positive surface charge) and SIT the sulfonatesilane coating (negative surface charge). NPs size morphology and coating thickness were evaluated by TEM analyses. The core/shell structure had a Fe_3_O_4_ magnetic core of 12.0 ± 2.0 nm, and a SiO_2_ shell thickness of 7.0 ± 1.5 nm, yielding a total particle diameter of 26.0 ± 2.9 nm. Figure 1 shows the characterization of bare NPs, while functionalized NPs are reported in Figure S1. The NPs size in solution was determined by the dynamic light scattering (DLS) measurements, confirming TEM results (Figure 1B, S1A, D). Z-Potential analysis showed that bare NPs and SIT passivated NPs had a negative surface charge (Figure 1C, S1C), while APTES modified NPs showed a positive surface charge (Figure S1F). NPs were characterized also in cell culture medium (DMEM), to evaluate the NPs stability and protein corona effects. In cell culture medium the hydrodynamic diameter increased for all the NPs (∼100 nm), regardless of the surface charge, indicating protein corona formation and presence of some NPs agglomeration, due to limited colloidal stability. This also leads to significant changes of nanoparticles surface charge with important consequences on NP/cell interaction and NPs uptake [28]. Actually, all the NPs acquired a negatively charged surface (about -35 mV) (Figure 2, S2).

In the cytotoxicity assays we investigated a wide range of NPs concentration (up to 5 nM) and incubation times (up to 96 h). The amount of Fe_3_O_4_/SiO_2_ NPs internalized by cells was determined through elemental analysis by inductively coupled plasma atomic emission spectroscopy (ICP-AES).

**Figure 3:**
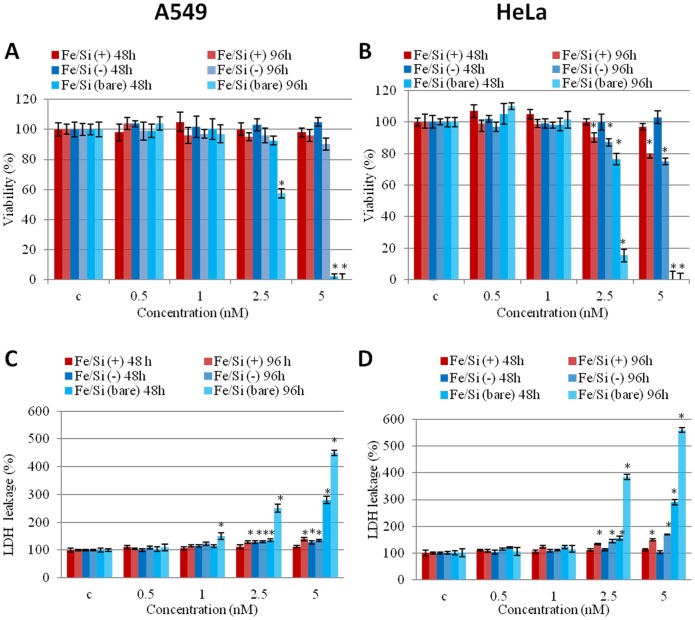
Effect of bare and passivated Fe_3_O_4_/SiO_2_ NPs on the viability and membrane damage in two cell lines (A549 and HeLa). (A, B) WST-8 proliferation assay and (C, D) LDH assay on A549 and HeLa cells incubated with increasing concentrations (0.5, 1, 2.5, 5 nM) of bare and passivated Fe_3_O_4_/SiO_2_ NPs at different times (48 and 96 h). C identifies the negative control in the absence of NPs. Viability of NPs-treated cells is expressed relative to non-treated control cells. As positive control (P), cells were incubated with 5% DMSO in WST-8 assay and 0.9% Triton X-100 in LDH assay (not shown). Data are reported as mean ±SD from three independent experiments; *P < 0.05 compared with control (n =  8).

Cell viability was evaluated by the WST-8 assay. Treatment with bare NPs showed a strong viability reduction at high NPs concentration (2.5, 5 nM) in both cell lines. On the other side, passivated NPs did not show any sign of toxicity, regardless of the surface charge (Figure 3A, B). In general, we observed that A549 cells were more resistant to NPs treatment. HeLa cells showed a slight toxicity also for passivated NPs at high NPs concentrations. LDH leakage assay was in close agreement with viability results. Bare NPs induced high levels of membrane damage, as opposed to both passivated NPs that induced only a slight increase of LDH levels at the maximum concentrations after 96 h (Figure 3C, D). Overall, the toxicity does not seem to be related to the size or to the surface charge of the NPs, but rather to the characteristic of the surface passivation.

**Figure 4:**
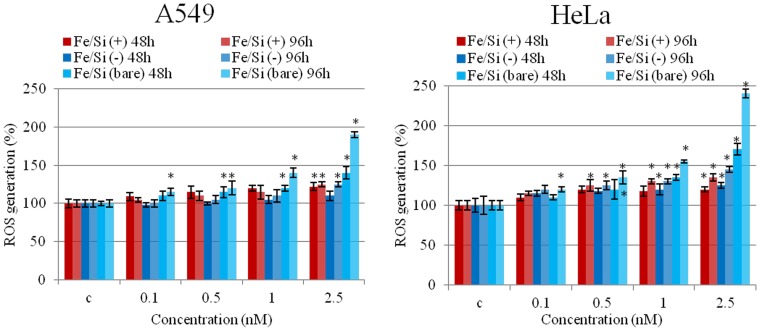
Effect of bare and passivated Fe_3_O_4_/SiO_2_ NPs on the ROS level in two cell lines (A549 and HeLa). Cells were treated with different concentration (0.1, 0.5, 1, 2.5 nM) of NPs for 48 and 96 h and ROS levels were evaluated by DCFH-DA assay. Percent ROS generation of nanoparticle-treated cells is expressed relative to non-treated control cells. As a positive control (P), cells were incubated with 500 µM H_2_O_2_ (not shown). Data are reported as mean ± SD from three independent experiments; *P < 0.05 compared with control (n =  8).

**Figure 5:**
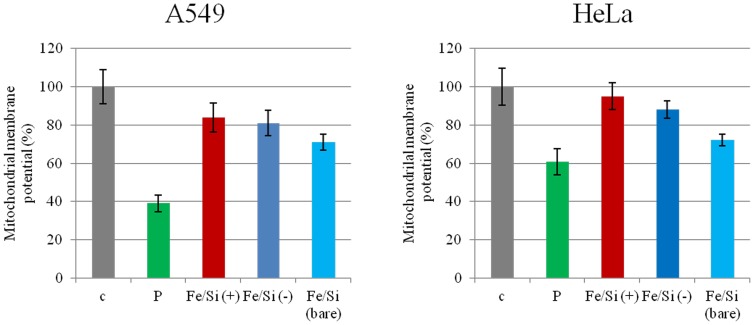
Effect of bare and passivated Fe_3_O_4_/SiO_2_ NPs on mitochondrial membrane potential (MMP) in two cell lines. Cells were treated with 2.5 nM of NPs for 48 h and percent of mitochondrial membrane potential of nanoparticle-treated cells was evaluated by JC-1 assay and was expressed relative to non-treated control cells. As a positive control (P) cells were incubated with 100 µM valinomycin. Data are reported as mean ± SD from three independent experiments; *P < 0.05 compared with control (n =  8).

**Figure 6:**
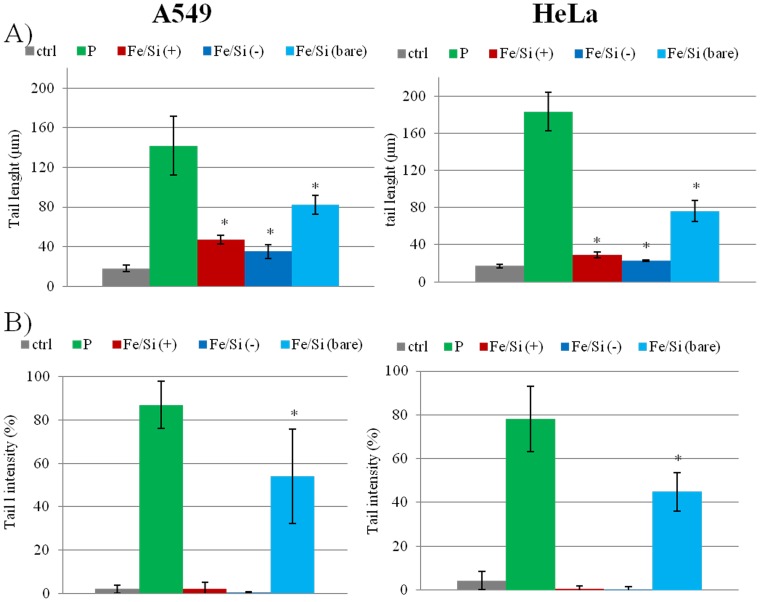
Effect of bare and passivated Fe_3_O_4_/SiO_2_ NPs on DNA damage. A549 and HeLa cells were treated with 2.5 nM of NPs for 48 h. DNA damage was evaluated through the comet assay by A) tail length and B) tail DNA intensity. Values shown are mean from 100 randomly selected comet images of each sample. As a positive control (P) cells were incubated with 500 µM H_2_O_2_. Data are reported as mean ± SD from three independent experiments; *P < 0.05 compared with control (n =  3).

**Figure 7:**
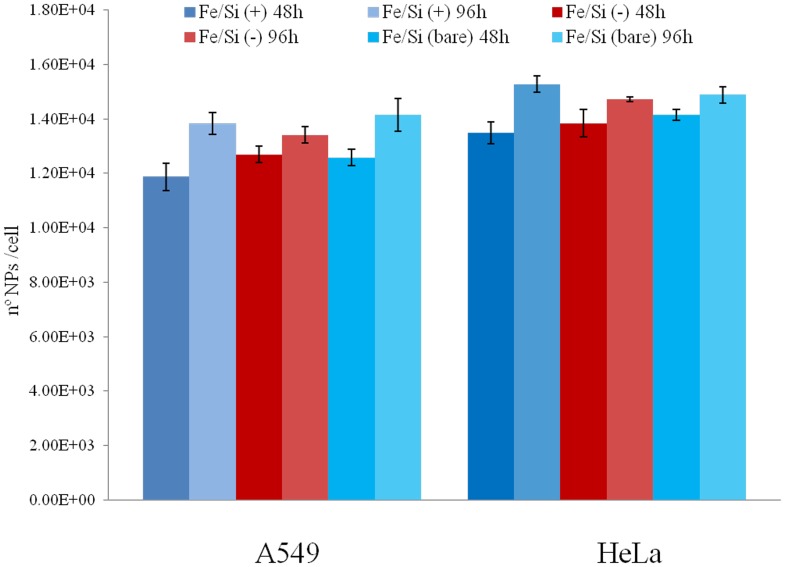
Cell uptake of bare and passivated Fe_3_O_4_/SiO_2_ NPs. Normalized internalization data for A549 and HeLa cells expressed as the number of nanoparticles internalized (determined by ICP-AES) per cell after 48 and 96 h of NPs incubation. Data are reported as mean ± SD from three independent experiments.

**Figure 8:**
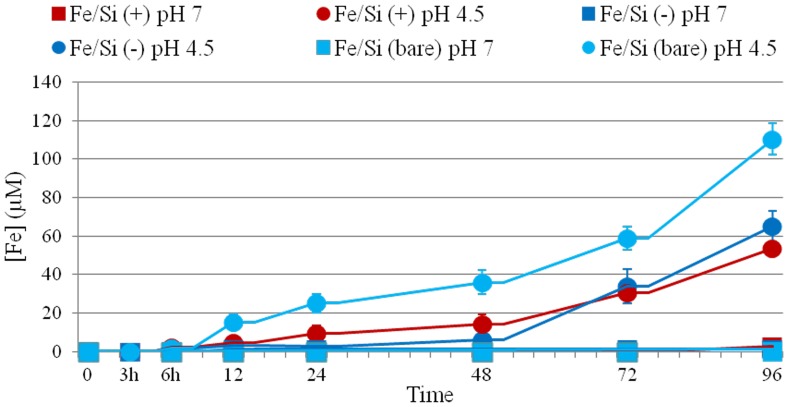
Effects of pH and surface functionalization on iron ions release from bare and passivated Fe_3_O_4_/SiO_2_ NPs. NPs degradation was evaluated both at pH 4.5 and pH 7 from 3 to 96 h. Neutral conditions were also probed in cell culture medium (DMEM, 10% FBS, pH 7.4), obtaining the same results (i.e., no detectable ion release).

**Figure 9:**
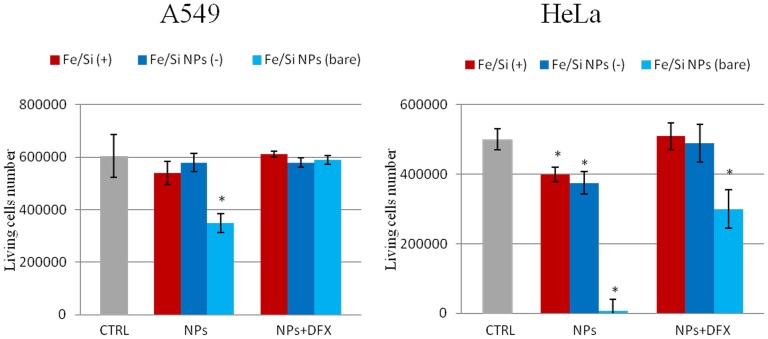
Effect of the iron chelator desferioxamine (DFX) on A549 and HeLa cells treated with NPs. Cells were treated with 100 µM of DFX and 5 nM of passivated NPs (positive and negative) or 2.5 nM of bare NPs for 96 h. Data are reported as mean ± SD from three independent experiments; *P < 0.05 compared with control (n =  8).

The ability of NPs to induce the production of intracellular oxidants in A549 and HeLa cells was assessed using DCF fluorescence as a reporter of ROS generation. In cells treated with bare NPs we observed a significant generation of ROS, especially after 96 h of incubation at high NPs concentration (2.5 nM). Passivated NPs induced the generation of low levels of ROS (no differences were found between the two surfaces charges) (Figure 4). Intracellular ROS production induces apoptosis and inflammation [29], [30], inhibits osteogenic differentiation of human mesenchymal stem cells [31], affects the actin cytoskeleton by modulating the Akt signaling pathway [32] resulting in a diminished cell proliferation [33] or increase in actin stress fiber formation [34]. ROS also alters mitochondria membrane potential [13] and damages DNA [35]. Thus, we assessed the mitochondrial membrane potentials of the two cells upon NPs treatment, using the JC-1 assay. The perturbation of mitochondrial membrane potential was more evident in cells treated with bare NPs, whereas passivated NPs induced only a slight not-statistically significant variation (Figure 5). Consistent with previous experiment, bare NPs were found to be significantly more toxic, than the passivated ones, which showed low levels of toxicity. In order to test the impact of this finding on genotoxicity, we performed the comet assay. The genotoxicity results were in good agreement with the cytotoxicity data, namely bare NPs strongly increased the levels of DNA damage, in terms of both tail length and DNA percentage in the tail, unlike the passivated NPs, showing values of DNA damage similar to the control (Figure 6). NPs DNA damage was also confirmed by the formation of characteristic comet morphology (Figure S3). In order to establish whether the different cytotoxicity effects could be due to different nanoparticles uptake we quantified the amount of Fe_3_O_4_/SiO_2_ NPs nanoparticles taken up per cell (Figure 7). This also clarifies the possible dependence of NPs uptake on surface charge [36], [37] and surface chemistry [38]. Experimental data confirmed that the uptake was very similar for the different NPs, regardless of their surface charge/chemistry, likely due to protein corona effects [24], [39], [40]. Hence, the different toxic effects of nanoparticles cannot be ascribed to different NPs uptake. Once discussed the cytotoxicity data, all consistent with each other, we go to their interpretation. We believe that the release of ferric ions from nanoparticles plays a key role in the NPs toxicity mechanism [21]. Iron ions released after the degradation of NPs into the acidic endolysosomal compartments could react with hydrogen peroxide produced by the mitochondria, inducing the generation of highly reactive hydroxyl radicals and ferric ions (Fe^3+^) through the Fenton reaction [41], [42]. We validated this idea evaluating the degradation of our Fe_3_O_4_/SiO_2_ NPs in an acidic medium mimicking the lysosomal environment [21] analyzing i) the role of surface passivation, ii) the effect of an iron chelator, the drug desferioxamine (DFX), which has a very high affinity and specificity for iron and it is currently used clinically to treat iron overload [43]. The release of iron ions from the NPs was assessed at pH 4.5 in a 20 mM citric medium and at pH 7 both in water and in cell culture medium (DMEM), at 37°C, up to 96 h. The amount of iron released from the nanoparticles was determined through elemental analysis. The kinetic of the nanoparticles dissolution was found to be strongly dependent on the pH and NPs surface coating. In fact, at pH 7, we could not detect iron release (this behavior was observed both in water and in DMEM). On the contrary, in acidic conditions, NPs significantly released iron in solution. In particular, bare NPs were the most prone to degradation, releasing 2-fold ions amount than passivated NPs (Figure 8). The presence of functional groups on the surface thus prevents the degradation *in-situ* of the NPs. The different ions release is thus responsible of the different toxicity/genotoxicity observed in previous experiments. To further validate this hypothesis (NPs toxicity mainly due to intracellular ions release) we performed experiments with iron chelator (DFX). The toxicity of bare NPs, which induced the highest decrease of cell viability, was strongly limited by the presence of DFX, emphasizing the importance of free iron (Figure 9). The passivation of NPs surface through the silanization agents creates an additional protective coating, which makes the silica shell less porous and more compact and stable [44]. This enhances NPs resistance to the acidic conditions of lysosomal environment, reducing the degradation process of the iron core and slowing down the ions release. It was demonstrated that DFX significantly reduced the ROS levels in cells treated with iron oxide NPs [41] and increased the viability of cells treated with iron ions [45]. We confirmed the close link between NPs surface passivation and cytotoxic effects by evaluating the viability of cells treated with Fe_3_O_4_/SiO_2_ NPs passivated with a lower amount of amine silanization agent. The presence of a lower amount of amino groups on NPs surface was confirmed by Zeta-Potential measurements (Figure S4). As expected, A549 and HeLa cells showed intermediate values of viability between more densely functionalized and bare NPs (Figure 10A), in close agreement with the iron ions release in acidic conditions (Figure 10B). We thus confirmed the fundamental role of NPs surface passivation in limiting the nanoparticles toxicity through the increase of resistance to lysosomal acidity, with consequent reduction of the iron ions release. The increased levels of free iron in the intracellular environment affect the ROS homeostasis. In fact, the Fe^2+^ ions could react with hydrogen peroxide and oxygen produced by the mitochondria to produce highly reactive hydroxyl radicals and ferric ions (Fe^3+^) via the Fenton reaction [46]:
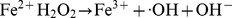



**Figure:**
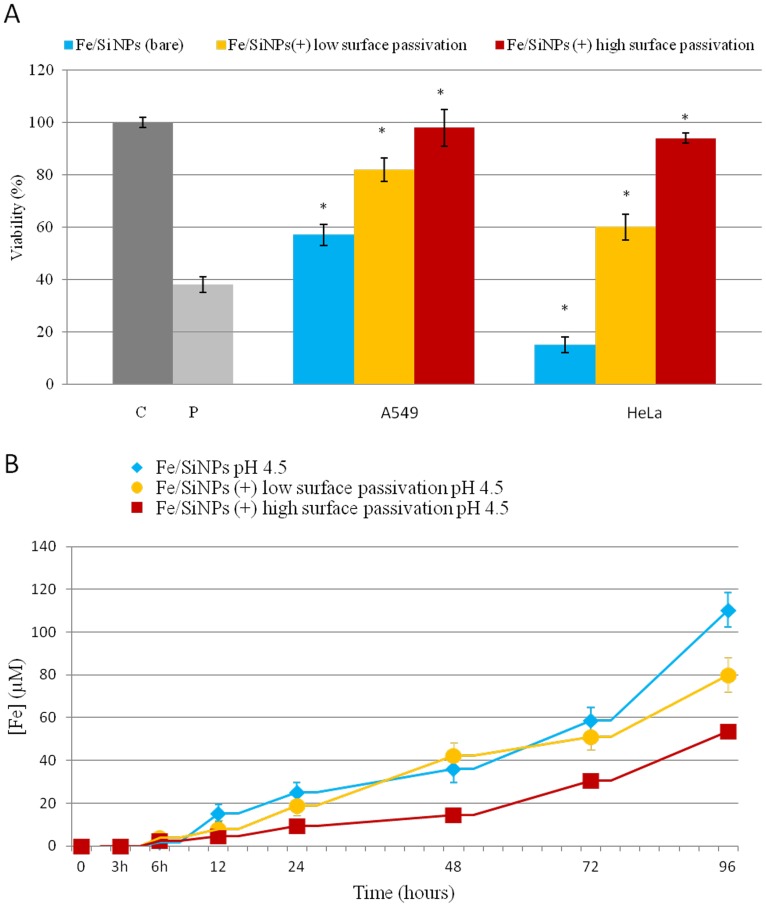
Effect of Fe_3_O_4_/SiO_2_ NPs surface passivation on the viability in two cell lines (A549 and HeLa). A) Viability of A549 and HeLa cells after 96h exposure to 2.5 nM of Fe_3_O_4_/SiO_2_ NPs with different amount of amine groups with WST-8 assay. Percent viability of nanoparticle-treated cells is expressed relative to non-treated control cells. As positive control (P), cells were incubated with 5% DMSO. Data are reported as mean ± SD from three independent experiments; *P < 0.05 compared with control (n =  8). B) Effect of NPs surface passivation on iron ions release from Fe_3_O_4_/SiO_2_ NPs. NPs degradation was evaluated both at pH 4.5 and 7 from 3 to 96 h.

Therefore, hydroxyl radicals generated by the free iron could damage DNA, proteins and lipids, leading to cell death. In conclusion, our study demonstrates that the intracellular dissolution of iron oxide nanoparticles causes cytotoxicity. The passivation of nanoparticles by surface functionalization may strongly improve their biocompatibility through enhancement of their resistance to lysosomal acidity. Therefore, surface engineering of iron oxide NPs may modulate the NPs dissolution kinetics, decreasing the overall toxicity creating a safe nanosystem for nanomedicine applications.

## Materials and Methods

### Synthesis of spherical iron oxide nanoparticles

In a typical synthesis of 10 nm Fe_3_O_4_, oleic acid (3.2 g, 12 mmol) and ODE (10 mL) were loaded in to a 50-mL three-necked flask connected to a reflux cooler and pumped to vacuum for 30 min at 120°C. After that the solution cooled down to room temperature and put under N_2_ flux. Then the precursor solution (Fe (CO)_5_ (0.597 g, 3 mmol) (dissolved in 2 mL of ODE) was injected and the mixture heated to the temperature of 320°C, with a heating rate of 20°C/min. The solution reaction was stirred vigorous in that temperature for 1.5 h. Then cooled down and exposed in air for 30 min at 130°C and then naturally cooled in room temperature. Finally, the NPs were collected by centrifuge and washed in a mixture of 2-propanol and acetone. The purified process carried out three times. At the end the NPs were stored in chloroform.

### Synthesis of Fe_3_O_4_/SiO_2_ nanoparticles (Fe/SiNPs)

The ternary microemulsion was composed of a surfactant, an organic solvent and water. 880 µL of Igepal (Sigma), 3.75 mL of cyclohexane (J.T. Baker), 60 µL of Fe_3_O_4_ NPs in chloroform were mixed together and stirred for 30 min. Then, 170 µL of water, 50 µL of TEOS (99%, Sigma Aldrich) and 60 µL of NH_4_OH (28–30%, Sigma Aldrich) were added to the microemulsion. The mixture was left to stir for 24 h. After the reaction was completed, acetone (J.T. Baker) was added to break the microemulsion. Nanoparticles were recovered by centrifugation (4500 rpm, 30 min, T =  25°C) and the surfactant and the unreacted molecules were washed out from the resultant precipitate of Fe_3_O_4_/SiO_2_ NPs sequentially, with butanol (Sigma Aldrich), iso-propanol (Carlo Erba reagents), ethanol (J.T. Baker) and water. The ultrasonic treatment was used to completely disperse the precipitate in the solvent and to remove the adsorbed molecules from the surface of the final product. The above mentioned conditions yielded Fe_3_O_4_/SiO_2_ NPs with a total diameter of 26 nm.

### Preparation of amine-modified Fe_3_O_4_/SiO_2_ nanoparticles

Fe_3_O_4_/SiO_2_ NPs were dispersed in freshly prepared 5% (v/v) solution of aminopropyltriethoxysilane (APTES, Sigma Aldrich) and 1 mM acetic acid (99.7%, Sigma Aldrich) and stirred for 60 min [47]_ENREF_44. After reaction, amine modified nanoparticles were separated by centrifugation (4500 rpm, 10 min), washed 5–6 times with acetone and water (1∶1). The nanoparticles were then redispersed in water.

### Preparation of sulfonated-modified Fe_3_O_4_/SiO_2_ nanoparticles

Surface modified Fe_3_O_4_/SiO_2_ NPs were prepared by condensation of 3-(trihydroxysilyl)-1-propanesulfonic acid (SIT) with surface silanol groups. NPs were dispersed in freshly prepared 10% (v/v) solution of SIT (Gelest). This clear suspension was left to react overnight while stirring. To eliminate unreacted SIT, the suspension was separated by centrifugation (4500 rpm, 10 min), washed 5–6 times with water. The nanoparticles were then redispersed in water.

### TEM analyses

Transmission electron microscope (TEM) images were recorded by a JEOL Jem 1011 microscope operating at an accelerating voltage of 100 kV. TEM samples were prepared by dropping a dilute solution of nanoparticles in water on carbon-coated copper grids (Formvar/Carbon 300 Mesh Cu).

### Dynamic Light Scattering (DLS) and ζ-Potential Measurements

Dynamic Light Scattering (DLS) and ζ-Potential measurements were performed on a Zetasizer Nano ZS90 (Malvern, USA) equipped with a 4.0 mW HeNe laser operating at 633 nm and an avalanche photodiode detector. Measurements were made at 25°C in aqueous solutions (pH 7).

DLS and ζ-potential measurements were made also in cell culture medium. Cell culture medium DMEM high glucose (Dulbecco's Modified Eagle Medium) was supplemented with 10% of Fetal Bovine Serum (FBS) (Gibco Invitrogen) as protein source, with 50 µM glutamine (Gibco), 1 mM sodium pyruvate (Gibco) 100 U/mL penicillin and 100 mg/mL streptomycin (Invitrogen).

Bare and passivated Fe_3_O_4_/SiO_2_ NPs were incubated at 37°C with the cell culture medium (DMEM 10% FBS), respectively DLS and ζ-potential measurements were taken after 96 h of incubation.

### Cell Culture

HeLa cells (human cervix carcinoma, IST cell bank, Interlab Cell Line Collection (ICLC) Accession number HTL95023) and A549 cells (human lung carcinoma, HTL03001) were routinely cultivated in high glucose DMEM with 50 µM glutamine, supplemented with 10% FBS, 100 U/mL penicillin and 100 mg/mL streptomycin. Cells were incubated in a humidified controlled atmosphere with a 95% to 5% ratio of air/CO_2_, at 37°C. Medium was changed every 3 days.

### WST-8assay

A549 and HeLa cells were seeded in 96 well microplates at a density of 5000 cells/well at final volume of 50 µl and incubated for 24 h in a humidified atmosphere at 37°C and 5% CO_2_ to obtain a subconfluent monolayer (60–70% of confluence). Fe_3_O_4_/SiO_2_ NPs were dispersed in cell culture medium to attain stock solutions and added at the single well obtaining a final Fe_3_O_4_/SiO_2_ NPs concentrations of 0.5, 1, 2.5, 5 nM in a final volume of 100 µl for each well. The metabolic activity of all cell cultures was determined after 48 and 96 hours of exposure to bare and passivated Fe_3_O_4_/SiO_2_ NPs, using a standard WST-8 assay (Sigma). Assays were performed following the procedure previously described in Brunetti *et al.* [48]. Data were expressed as mean ± SD. Differences in cell proliferation (WST-8) between cells treated with Fe_3_O_4_/SiO_2_ NPs and the control were considered statistically significant performing a *t*-student test with a *p*-value < 0.05.

### LDH assay

HeLa and A549 cells were seeded in black 96 well microplates (Constar) and treated with bare and passivated Fe_3_O_4_/SiO_2_ NPs at concentrations of 0.5, 1, 2.5, 5 nM, following the procedures reported for the WST-8 assay. After 48 and 96 hours of cells-NPs interaction, the lactate dehydrogenase (LDH) leakage assay was performed onto microplates by applying the CytoTox- ONE Homogeneous Membrane Integrity Assay reagent (Promega), following the manufacturer's instructions and the procedure described in Brunetti *et al.* [48]. Data were expressed as mean ± SD. Differences in LDH leakage between cells treated with Fe_3_O_4_/SiO_2_ NPs and controls were considered statistically significant performing a *t*-student test with a *p-*value < 0.05.

### DCF assay

HeLa and A549 cells were seeded in 96-well microplates and treated with bare and passivated Fe_3_O_4_/SiO_2_ NPs at a final concentration of 0.1, 0.5, 1, 2.5 nM. After 48 and 96 hours of cells-NPs interaction the DCF-DA (2′,7′-Dichlorofluorescein diacetate, Sigma) assay was performed onto microplates following the procedure reported by Malvindi *et al.* [23]. Data were expressed as mean ± SD. Differences in ROS generation between cells treated with Fe_3_O_4_/SiO_2_ NPs and controls were considered statistically significant performing a *t*-student test with a *p*-value < 0.05.

### JC-1 assay

HeLa and A549 cells were seeded in black 96 well microplates (Constar) and treated with bare and passivated Fe_3_O_4_/SiO_2_ NPs at concentrations of 0.5, 1, 2.5, 5 nM, following the procedures reported for the WST-8 assay. After 48 and 96 hours of cells-NPs interaction, the mitochondrial membrane potential assay (Molecular probes JC-1, Invitrogen) was performed onto microplates, following the manufacturer's instructions by using Fluo Star Optima (BMG LABTECH) microplates reader. On the day of the experiments, after removing the medium, the cells in the plates were washed with PBS buffer and then incubated with 2.5 µg/mL JC-1 in the loading medium (DMEM 1% FBS). After loading medium was removed, the cells were washed and incubated with PBS buffer and the fluorescence of the cells from each well was measured and recorded. JC-1 loaded cells were placed in a CytoFluor Series 4000 multiwell fluorescence plate reader (PerSeptive Biosystems Inc., Framingham, MA, USA) with temperature maintained at 37°C. The excitation filter was set at 520–570 nm and the emission filter was set at 570–610 nm. As negative controls, we applied the same assay onto untreated cells. Results are normalized with respect to negative controls (expressed as 100%). As a positive control for cytotoxicity, cells were incubated with 100 µM valinomycin. Data were expressed as mean ± SD. Differences in mitochondrial membrane potential between cells treated with Fe_3_O_4_/SiO_2_ NPs and controls were considered statistically significant performing a *t*-student test with a *p*-value < 0.05.

### The Comet assay (single cell gel electrophoresis)

In the Comet assay, A549 and HeLa cells were exposed to 5 nM of bare and passivated Fe_3_O_4_/SiO_2_ NPs for 48 h, at density of 5 × 10^4^ in each well of 12-well plates in a volume of 1.5 mL. After treatments, cells were centrifuged and suspended in 10 µl of PBS at concentration of 1000 cells/µl. The cell pellets were mixed gently with 75 µl of 0.75% low-melting-point agarose (LMA) and then and then layered onto microscope slides precoated with 1% normal melting agarose (NMA) and dried at room temperature. Two gels were mounted on each slide and covered with a coverslip. After agarose solidification (for 10 min at 4°C), the cover slips were removed and and the slides were immersed in a cold fresh Lysis solution (2.5 M NaCl, 100 mM EDTA, 10 mM Tris, 1% Triton X-100, and 10% DMSO, pH 10) for 1 h at 4°C in a dark chamber. Subsequently, the slides were immersed in an alkaline solution (300 mM NaOH, 1 mM Na_2_EDTA, pH 13) for 20 min to allow for unwinding of the DNA. The electrophoresis was carried out in the same buffer for 25 min at 25 V and 300 mA (0.73 V/cm). After electrophoresis, cellular DNA was neutralized by successive incubations in a neutralized solution (0.4 M Tris-HCl, pH 7.5) for 5 minutes at room temperature. The slides were stained with 80 µl SYBR Green I Nucleic Acid Gel Stain solution (Invitrogen) in the dark at room temperature for 1 hour. Comets derived from single cells were photographed under a Leica fluorescence microscope and head and tail DNA intensity of each comet were quantified using Comet IV program (Perceptive Instruments). Differences in DNA damage between cells treated with Fe_3_O_4_/SiO_2_ NPs and controls were considered statistically significant performing a *t*-student test with a *p*-value < 0.05.

### Determination of the Intracellular Uptake of Fe_3_O_4_/SiO_2_ nanoparticles

To estimate the intracellular Fe concentration and hence the intracellular nanoparticles uptake, 10^5^ cells were seeded in 1 mL of medium in each well (3.5 cm in diameter) of a 6-well plate. After 24 h of incubation at 37°C, the medium was replaced with fresh medium containing the nanoparticles at a concentration of 2.5 nM. After 48 and 96 h of incubation at 37°C, the medium was removed, the cells were washed three times with PBS (pH 7.4), trypsinized, and counted using a cell-counting chamber. Then, the cell suspensions were digested overnight in 1 mL of concentrated HCl/HNO_3_ 3∶1 (v/v), diluted to 5 mL with ultrapure water, and the resulting solution was directly analyzed to evaluate the intracellular Fe concentration through elemental analysis and normalized to the number of cells. Elemental analysis was carried out by inductively coupled plasma atomic emission spectroscopy (ICP-AES) with a Varian Vista AX spectrometer.

### Measurements of NPs ions release

The evaluation of NPs ions release was performed at 37°C both in acidic conditions (sodium citrate buffer, pH 4.5), an acidic medium mimicking the lysosomal environment [20], [21] and in neutral conditions (ultrapure water or cell culture medium, pH 7). The citrate buffer was prepared by mixing appropriate volumes of 20 mM aqueous solutions of citric acid and sodium citrate monobasic to achieve the final desired pH. Adequate volumes of stock nanoparticle suspensions were diluted in buffer and in water in order to achieve the final NPs concentrations of 40 nM. The nanoparticles were then incubated at 37°C. The ions release was analyzed over time at 24, 48, 72 and 96 hours. At each time point, the NPs were separated from the rest of solution through centrifugation at 13000 rpm for 1 hour. Solutions were collected and digested by the addition of a solution of HCl/HNO_3_ 3∶1 (v/v), and the amount of free ions was measured by ICP-AES (Inductively Coupled Plasma Atomic Emission Spectrometer).

### Effect of Desferrioxamine (DFX) on living cells number

A549 and HeLa cells were seeded in 1 mL of medium in each well (3.5 cm in diameter) of a 6 well plate at a density of 10^5^ cells per well and incubated for 24 h in a humidified atmosphere at 37°C and 5% CO_2_ to obtain a subconfluent monolayer (60–70% of confluence). After 24 h of incubation, the medium was removed, the cells were washed three times with PBS (pH 7.4), and replaced with fresh medium. To study the effect of iron chelator on cell viability, cells were incubated with DFX (100 µM) and a NPs suspension of Fe_3_O_4_/SiO_2_ NPs (5 nM). As controls, cells were incubated both with Fe_3_O_4_/SiO_2_ NPs (5 nM) without iron chelator and only with the iron chelator (100 µM). After 96 h of incubation at 37°C, the medium was removed the cells were washed three times with PBS (pH 7.4), trypsinized, and counted using a cell-counting chamber. Differences in living cells number between cells treated with Fe_3_O_4_/SiO_2_ NPs and controls were considered statistically significant performing a *t*-student test with a *p*-value < 0.05.

### Statistical analyses

GraphPad Prism 5 statistical analysis software was used for all statistical analyses performed in this work (GraphPad Prism version 5.00 for Windows, GraphPadSoftware, San Diego, California, USA). Data were analyzed by one-way ANOVA and compared to the corresponding control by the Bonferroni post-test. Differences between treated samples and controls were considered statistically significant for P-values <0.05(*) and non-significant for P-values >0.05.

## Supporting Information

Figure S1 **Characterization of (negatively and positively charged) passivated Fe_3_O_4_/SiO_2_ NPs in water.** (A, D) TEM images, (B, E) Dynamic light scattering, (C, F) ζ-Potential measurements.
(TIF)

Figure S2 **Characterization of (negatively and positively charged) passivated Fe_3_O_4_/SiO_2_ NPs in cell culture medium.** ζ-potential and Dynamic light scattering measurements of negative (A, B) and positive (C, D) Fe3O4/SiO2 NPs suspended in DMEM culture medium for 96 h.
(TIF)

Figure S3 **Effect of bare and passivated Fe_3_O_4_/SiO_2_ NPs on DNA damage.** Representative images of positive, negative and bare Fe3O4/SiO2 NPs effects on DNA damage in A549 and HeLa cells determined by comet assay.
(TIF)

Figure S4 **Characterization of Fe_3_O_4_/SiO_2_ NPs passivated with a low amount of passivation agent.** ζ-potential measurements in water of Fe3O4/SiO2 NPs passivated.
(TIF)
