# Supplementary figures and images for: Toxicity Assessment of Silica Coated Iron Oxide Nanoparticles and Biocompatibility Improvement by Surface Engineering
Source: PLoS One. 2014 Jan 21;9(1):e85835. doi: 10.1371/journal.pone.0085835 (PMC3897540; doi:10.1371/journal.pone.0085835)

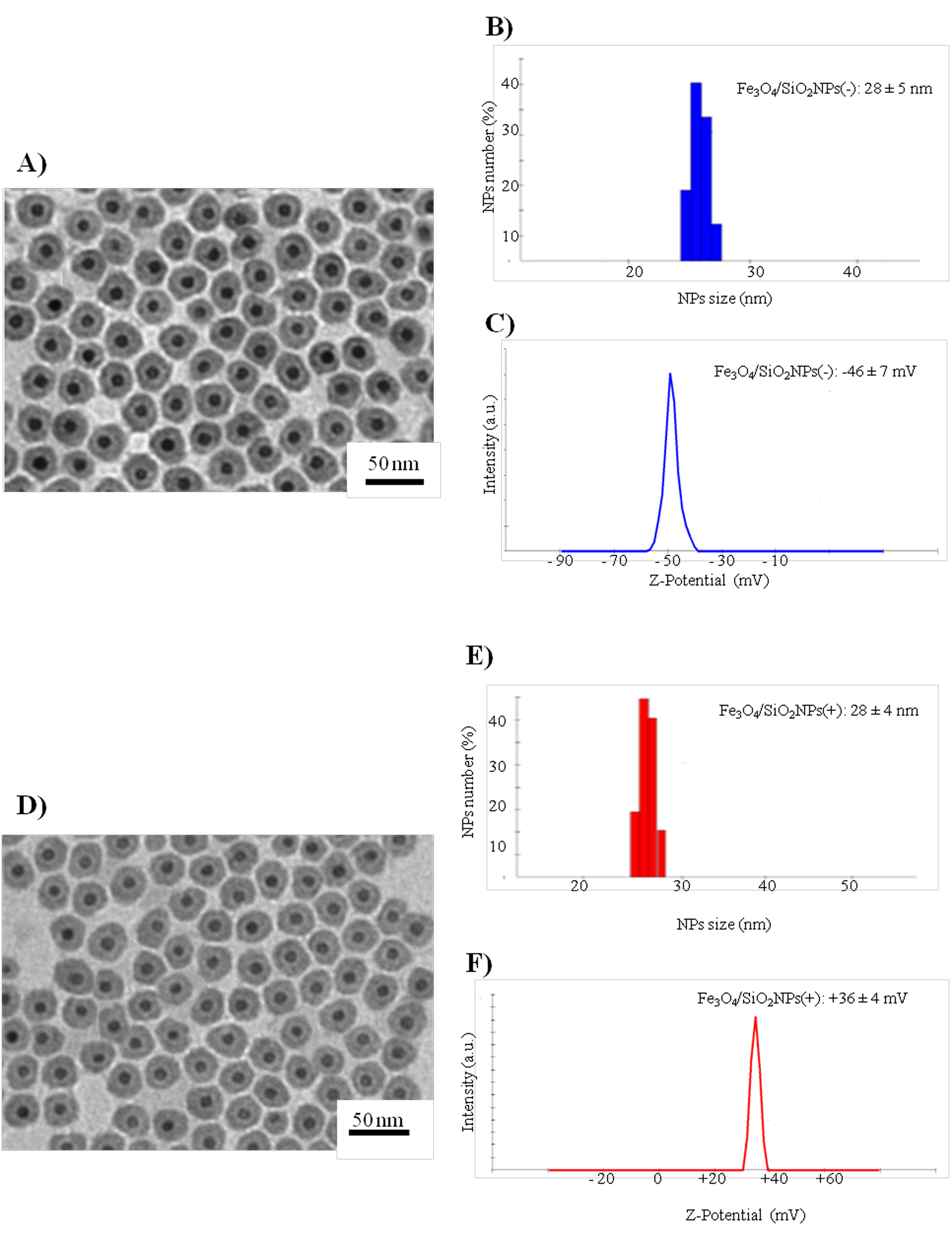

Supplement: Figure S1 — Characterization of (negatively and positively charged) passivated Fe3O4/SiO2 NPs in water. (A, D) TEM images, (B, E) Dynamic light scattering, (C, F) ζ-Potential measurements. (TIF) [file pone.0085835.s001.tif]

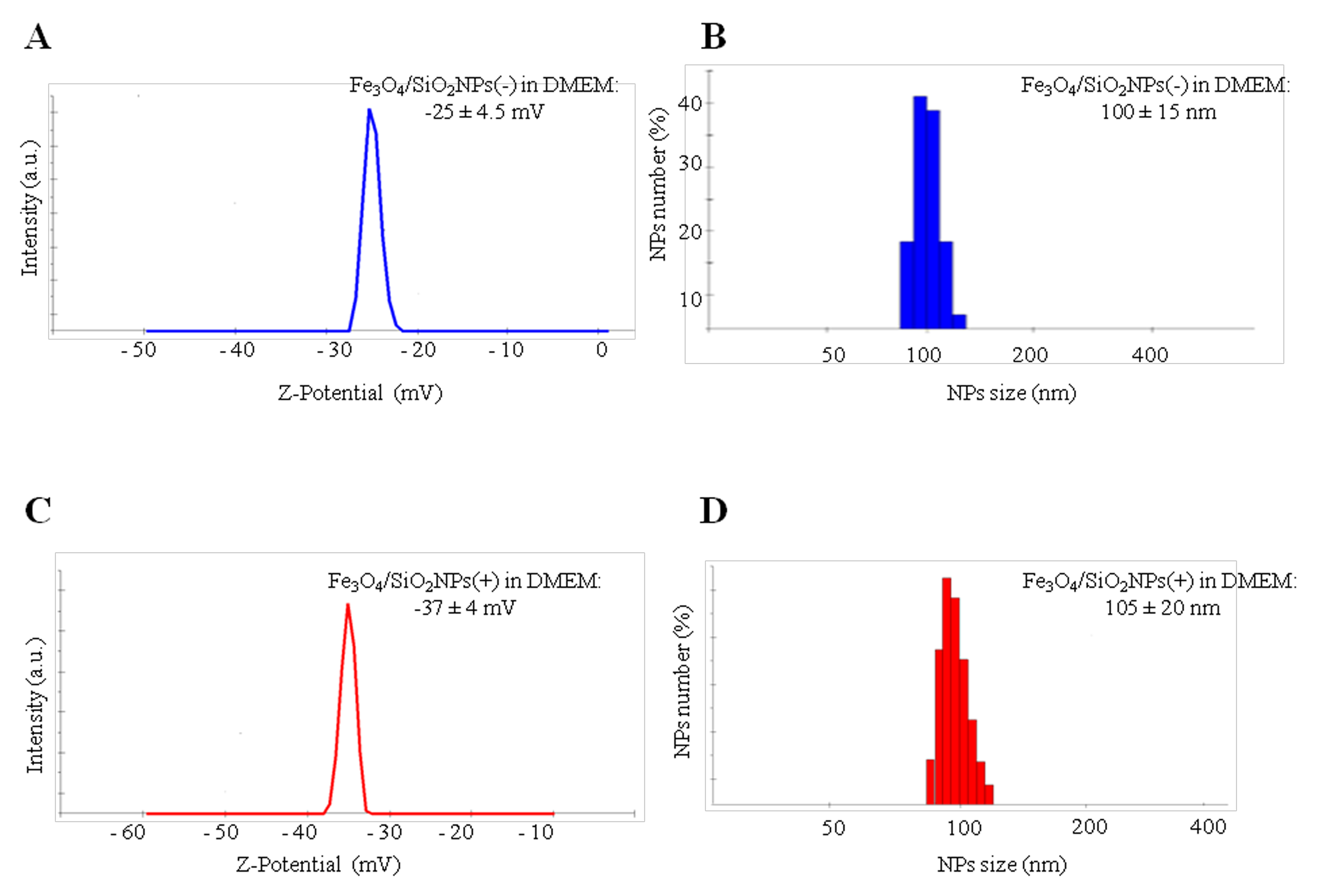

Supplement: Figure S2 — Characterization of (negatively and positively charged) passivated Fe3O4/SiO2 NPs in cell culture medium. ζ-potential and Dynamic light scattering measurements of negative (A, B) and positive (C, D) Fe3O4/SiO2 NPs suspended in DMEM culture medium for 96 h. (TIF) [file pone.0085835.s002.tif]

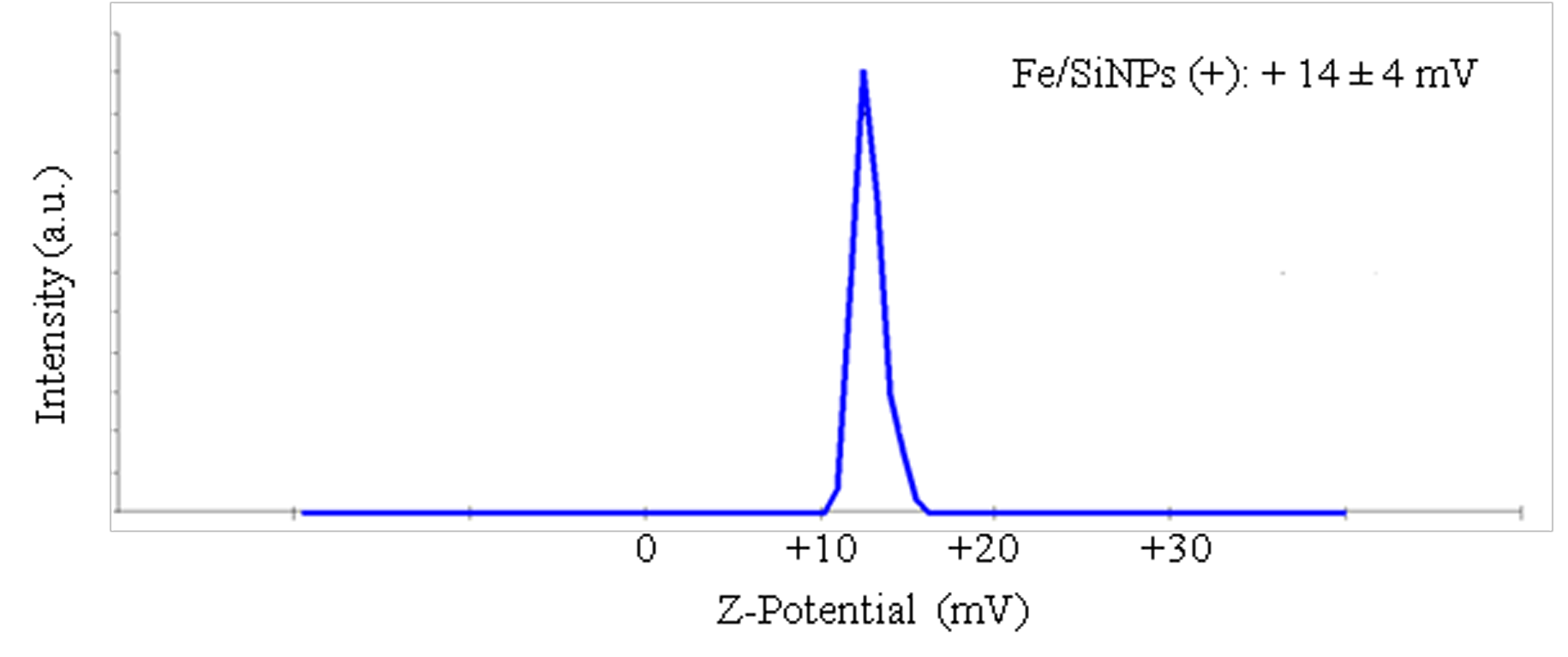

Supplement: Figure S4 — Characterization of Fe3O4/SiO2 NPs passivated with a low amount of passivation agent. ζ-potential measurements in water of Fe3O4/SiO2 NPs passivated. (TIF) [file pone.0085835.s004.tif]
